# Effects of ****ω****-3 PUFAs Supplementation on Myocardial Function and Oxidative Stress Markers in Typical Rett Syndrome

**DOI:** 10.1155/2014/983178

**Published:** 2014-01-12

**Authors:** Silvia Maffei, Claudio De Felice, Pierpaolo Cannarile, Silvia Leoncini, Cinzia Signorini, Alessandra Pecorelli, Barbara Montomoli, Stefano Lunghetti, Lucia Ciccoli, Thierry Durand, Roberto Favilli, Joussef Hayek

**Affiliations:** ^1^Department of Cardiology, University Hospital Azienda Ospedaliera Universitaria Senese (AOUS), Viale M. Bracci 16, 53100 Siena, Italy; ^2^Neonatal Intensive Care Unit, University Hospital AOUS, Viale M. Bracci 16, 53100 Siena, Italy; ^3^Department of Medical Biotechnologies, University of Siena, Strada delle Scotte 4, 53100 Siena, Italy; ^4^Department of Molecular and Developmental Medicine, University of Siena, Via A. Moro 2, 53100 Siena, Italy; ^5^Child Neuropsychiatry Unit, University Hospital AOUS, Viale M. Bracci 16, 53100 Siena, Italy; ^6^Institut des Biomolécules Max Mousseron (IBMM), UMR 5247, CNRS/UM1/UM2, BP 14491 34093, Montpellier, Cedex 5, France

## Abstract

Rett syndrome (RTT) is a devastating neurodevelopmental disorder with a 300-fold increased risk rate for sudden cardiac death. A subclinical myocardial biventricular dysfunction has been recently reported in RTT by our group and found to be associated with an enhanced oxidative stress (OS) status. Here, we tested the effects of the naturally occurring antioxidants **ω**-3 polyunsaturated fatty acids (**ω**-3 PUFAs) on echocardiographic parameters and systemic OS markers in a population of RTT patients with the typical clinical form. A total of 66 RTT girls were evaluated, half of whom being treated for 12 months with a dietary supplementation of **ω**-3 PUFAs at high dosage (docosahexaenoic acid ~71.9 ± 13.9 mg/kg b.w./day plus eicosapentaenoic acid ~115.5 ± 22.4 mg/kg b.w./day) versus the remaining half untreated population. Echocardiographic systolic longitudinal parameters of both ventricles, but not biventricular diastolic measures, improved following **ω**-3 PUFAs supplementation, with a parallel decrease in the OS markers levels. No significant changes in the examined echocardiographic parameters nor in the OS markers were detectable in the untreated RTT population. Our data indicate that **ω**-3 PUFAs are able to improve the biventricular myocardial systolic function in RTT and that this functional gain is partially mediated through a regulation of the redox balance.

## 1. Introduction 

Rett syndrome (RTT) is a genetically determined, neurodevelopmental disorder with autistic features [1, 2]. Although relatively rare, RTT represents the second most common cause of severe intellective disability in the female gender. To date, the disease has been classified into a typical form and three main atypical variants, that is, preserved speech, early seizure, and congenital.

In up to 95% of cases, RTT is caused by *de novo *mutation in the X-linked gene encoding MeCP2, a protein known to either activate or repress several transcriptional genes [3, 4].

Cumulating evidence indicates that RTT, for a long time considered exclusively a disease of the brain, is actually a systemic disease with involvement of several organs besides the brain, including autonomic nervous system, lung, bone, and heart [5–8]. Girls affected by typical RTT show a 300-fold increased risk for sudden cardiac death as compared to general population (about 26% of all deaths are sudden and of unknown cause [9]), although a satisfactory explanation for the association is still missing. In the lack of evidence for an increased prevalence of congenital heart defects, the attention of several authors has been focused on the presence of cardiac dysautonomia and rhythm abnormalities. In particular, a prolonged QT interval, an indicator of a repolarization abnormality and a well-established risk factor for sudden cardiac death [10], is reported in nearly 20% of patients with Rett syndrome [9, 11–13]. Abnormally persistent sodium currents have been reported in cardiomyocytes from *Mecp*2^Null/y^ mice and found to be normalized by the sodium channel-blocking antiepileptic drug phenytoin, which strongly suggests a brain-heart link as a possible explanation for the increased risk of sudden death in RTT [13]. However, our recent observation of a subclinical myocardial biventricular dysfunction in a large series of typical and atypical RTT patients may add new perspectives to the heart involvement in this neurodevelopmental disease [14].

Evidence of enhanced oxidative stress (OS) and, in particular, lipid peroxidation has been well established by our group in blood samples from patients with RTT and recently confirmed in primary skin fibroblasts cultures [15–21]. However, the molecular pathways linking the *MeCP2 *gene mutation to the OS derangement remain to be explored and, in particular, whether the nature of the relationship between *MeCP2 *gene mutation and abnormal redox homeostasis is causal or correlational remains currently unclear [22].

At the same time, experimental models have shown that OS is detrimental for myocardial function [23, 24]. Therefore, we speculate that OS may play a role in the myocardial dysfunction of RTT patients.

Omega-3 polyunsaturated fatty acids (*ω*-3 PUFAs) are natural molecules with a wide range of physiological functions on multiple tissues including the heart. In particular, *ω*-3 PUFAs are able to affect a myriad of molecular pathways, including alteration of physical and chemical properties of cellular membranes, direct interaction with and modulation of membrane channels and proteins, regulation of gene expression via nuclear receptors and transcription factors, changes in eicosanoid profiles, and conversion of *ω*-3 PUFAs to bioactive metabolites [25].

*ω*-3 PUFAs have gained increasing attention in the prevention of cardiovascular disease, although their biological effects and molecular mechanisms are highly debated [25].

In previous studies, we have demonstrated that supplementation of *ω*-3 PUFAs moderately reduces clinical severity and significantly reduces the levels of several OS biomarkers in the blood of RTT patients [17, 19, 26].

The aim of the present study was to assess the effects of 12 months of dietary supplementation with high-dose *ω*-3 PUFAs on the RTT-related subclinical myocardial dysfunction.

## 2. Methods 

### 2.1. Patients

In this study, a total of 66 RTT patients (mean age 12.7 ± 9.1 years) with typical presentation and demonstrated *MeCP2 *mutation were enrolled (Table 1) [27]. RTT diagnosis and inclusion/exclusion criteria were based on the recently revised RTT nomenclature consensus [28, 29]. RTT clinical severity was assessed using the clinical severity score (CSS), a validated clinical rating specifically designed for RTT, based on 13 individual ordinal categories measuring clinical features common in RTT [28]. All the patients were admitted to Child Neuropsychiatric Unit, University Hospital Azienda Ospedaliera Universitaria Senese (Head Dr. Joussef Hayek). Blood samplings in the patients' group were performed during the routine follow-up study at hospital admission. Sampling from the control group was carried out during routine health checks, sports, or blood donations obtained during the periodic clinical checks. The study was conducted with the approval of the Institutional Review Board and all informed consents were obtained from either the parents or the legal tutors of the enrolled patients.

### 2.2. Study Design

The experimental design was single centre, single blind, and randomized. Patients were randomized at admission (*n* = 33 treated, mean age at supplementation time zero: 13.0 ± 8.6 years; *n* = 33 untreated, mean age at time zero: 12.4 ± 9.3 years) to either oral supplementation with *ω*-3 PUFAs oil for twelve months or no treatment.

Administered *ω*-3 PUFAs were in the form of fish oil (Norwegian Fish Oil AS, Trondheim, Norway, Product Number HO320-6; Italian importer: Transforma AS Italia, Forlì Italy; Italian Ministry Registration Code: 10 43863-Y) at a dose of 5 mL twice daily, corresponding to docosahexaenoic acid (DHA) 71.9 ± 13.9 mg/kg b.w./day and eicosapentaenoic acid (EPA) 115.5 ± 22.4 mg/kg b.w./day, with a total *ω*-3 PUFAs 242.4 ± 47.1 mg/kg b.w./day. Use of EPA plus DHA in RTT was approved by the AOUS Ethical Committee.

All the subjects, included patients, examined in this study were following a standard Mediterranean diet.

### 2.3. Echocardiography

The study was performed using a commercially available echocardiography equipment (Philips IE 33 Vision 2009, qLAB 7.0 software; 5 and 8 MHz transducers) as previously reported [14]. Briefly, two-dimensional right and left chambers quantification (areas and volumes), left ventricle ejection fraction (Simpson's method), and pulmonary arterial systolic pressure (PASP) were estimated. Mitral flow velocities (*E* wave, *A* wave, and *E*/*A* ratio) were recorded using pulsed wave (PW) Doppler on the mitral valve. The evaluation of left and right ventricular longitudinal systolic function was performed by (a) mitral annular plane systolic excursion (MAPSE) and tricuspid annular plane systolic excursion (TAPSE), using M-mode, and (b) systolic (*S*′) and early diastolic (*E*′) peak velocities, using PW tissue Doppler imaging (TDI) of the lateral (lat) and septal (sep) mitral annulus for left ventricle (LV) and of tricuspidal annulus for right ventricle (RV) in four-chamber apical view. The *E*/*E*′_lat_ ratios were determined as surrogate of LV filling pressures.

In order to reduce operator-dependent bias, all measures were performed by two operators, blinded for clinical and therapeutical data of RTT group.

### 2.4. Blood Sampling

Blood sampling was carried out in all subjects at around 8.00 am after the overnight fast. For the *ω*-3 PUFAs treated group, blood sampling was performed the day before starting the supplementation and the day after the end of the selected 12-month period.

Blood was collected in heparinized tubes, and all manipulations were carried out within 2 h after collection. Blood samples were centrifuged at 2,400 g for 15 min at room temperature. The platelet poor plasma was saved, and the buffy coat was removed by aspiration. The erythrocytes were washed twice with physiological solution, resuspended in ringer solution (125 mM NaCl, 5 mM KCl, 1 mM MgSO_4_, 32 mM HEPES, 5 mM glucose, 1 mM CaCl_2_), pH 7.4 as a 50% (vol/vol) suspension, and then used for the determination of erythrocyte non protein-bound iron (NPBI).

Plasma was used for free isoprostanes (F_2_-isoprostanes, F_2_-IsoPs, and F_4_-neuroprostanes, F_4_-NeuroPs), 4-hydroxynonenal protein adducts (4-HNE PAs), and NPBI determinations. For all isoprostane determinations, butylated hydroxytoluene (BHT) (90 *μ*M) was added to plasma as an antioxidant and stored under nitrogen at −70°C until analysis.

### 2.5. Intraerythrocyte and Plasma NPBI

NPBI is a pro-oxidant factor, associated with hypoxia, hemoglobin oxidation, and subsequent heme iron release [30]. Intraerythrocyte and plasma NPBI were determined as a desferrioxamine (DFO)-iron complex by high-performance liquid chromatography, as previously reported [15].

### 2.6. Plasma Isoprostanes

Isoprostanes are considered the gold standard for the OS *in vivo *evaluation [31, 32]. Specifically, F_2_-IsoPs are the end products of arachidonic acid oxidation, a polyunsaturated fatty acid which is abundant in both brain grey and white matter. F_4_-NeuroPs are the end products of docosahexaenoic acid, abundant in neuronal membranes. Plasma F_2_-IsoPs and F_4_-NeuroPs were determined by a gas chromatography/negative ion chemical ionization tandem mass spectrometry (GC/NICI-MS/MS) analysis after solid-phase extraction and derivatization steps [33, 34].

For F_2_-IsoPs, the measured ions were the product ions at m/z 299 and m/z 303 derived from the [M-181]^−^ precursor ions (m/z 569 and m/z 573) produced from 15-F_2t_-IsoPs and PGF_2*α*_-d_4_, respectively [34]. For F_4_-NeuroPs, the measured ions were the product ions at m/z 323 and m/z 303 derived from the [M-181]^−^precursor ions (m/z 593 and m/z 573) produced from oxidized DHA and the PGF_2*α*_-d_4_, respectively [19].

### 2.7. Plasma 4-HNE PAs

Plasma 4-hydroxynonenal protein adducts (4-HNE PAs) are markers of protein oxidation due to aldehyde binding from lipid peroxidation sources [35]. Western blot protocols were performed as previously described [18].

Plasma proteins (30 *μ*g protein) were resolved on 4–20% SDS-PAGE gels (Lonza Group Ltd., Switzerland) and transferred onto a hybond ECL nitrocellulose membrane (GE Healthcare Europe GmbH, Milan, Italy). After blocking in 3% nonfat milk (BioRad, Hercules, CA, USA), the membranes were incubated overnight at 4°C with goat polyclonal anti-4-HNE adduct antibody (code AB5605; Millipore Corporation, Billerica, MA, USA). Following washes in TBS Tween and incubation with specific secondary antibody (mouse anti-goat horseradish peroxidase-conjugated, Santa Cruz Biotechnology Inc., CA, USA) for 1 h at RT, the membranes were incubated with ECL reagents (BioRad, Hercules, CA, USA) for 1 min. The bands were visualized by autoradiography.

Quantification of the significant bands was performed by digitally scanning the amersham hyperfilm ECL (GE Healthcare Europe GmbH, Milan, Italy) and measuring immunoblotting image densities with ImageJ software.

### 2.8. Statistical Analysis

All variables were tested for normal distribution (D'Agostino-Pearson test). Differences between groups were evaluated using independent-sample *t*-test (continuous normally distributed data), Mann-Whitney rank sum test (continuous nonnormally distributed data), and Kruskal-Wallis test. Associations between variables were tested by nonparametric univariate regression analysis. Two-tailed *P* values of less than 0.05 were considered significant. The MedCalc version 12.1.4 statistical software package (MedCalc Software, Mariakerke, Belgium) was used.

## 3. Results 

### 3.1. Effect of 12-Month *ω*-3 PUFA Supplementation on Myocardial Function

All patients of the *ω*-3 PUFAs arm of the study completed the 12-month supplementation and no side effects were observed. Phenotypical severity, biometric data, and bone densitometry estimates as well as serum 25-OH vitamin D levels were found to be comparable between the *ω*-3 PUFAs-supplemented and unsupplemented RTT subpopulations (Table 1) [27].

**Figure 1:**
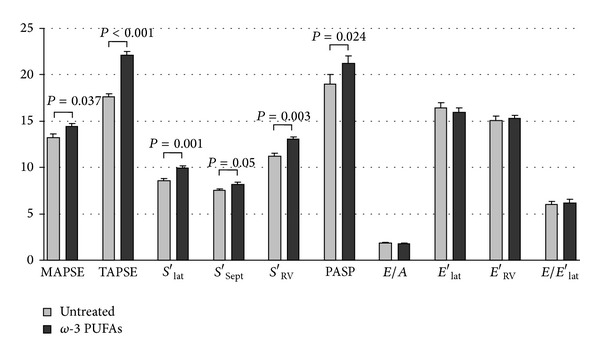
Dietary *ω*-3 PUFAs supplementation for 12 months significantly improves biventricular systolic longitudinal parameters in girls with typical Rett syndrome. MAPSE: mitral annular plane systolic excursion; TAPSE: tricuspid annular plane systolic excursion; *S*′_lat_: peak systolic velocity of lateral mitral annulus; *S*′_sep_: peak systolic velocity of septal mitral annulus; *S*′_RV_: peak systolic velocity of tricuspid annulus of right ventricle; PASP: pulmonary arterial systolic pressure; *E*/*A*: ratio between peak early diastolic mitral flow (*E*) and peak late diastolic mitral flow (*A*); *E*′_lat_: peak early diastolic velocity of lateral mitral annulus; *E*′_RV_: peak early diastolic velocity of tricuspid annulus of right ventricle; *E*/*E*′_lat_: this ratio indirectly estimates left ventricle end-diastolic filling pressure.

Following *ω*-3 PUFAs (EPA plus DHA) supplementation, significant improvements in systolic longitudinal parameters of both ventricles were observed (Figure 1), along with increased PASP. On the other hand, no significant changes in the echocardiographic parameters were detectable in the untreated RTT patients.

### 3.2. OS Markers

**Figure 2:**
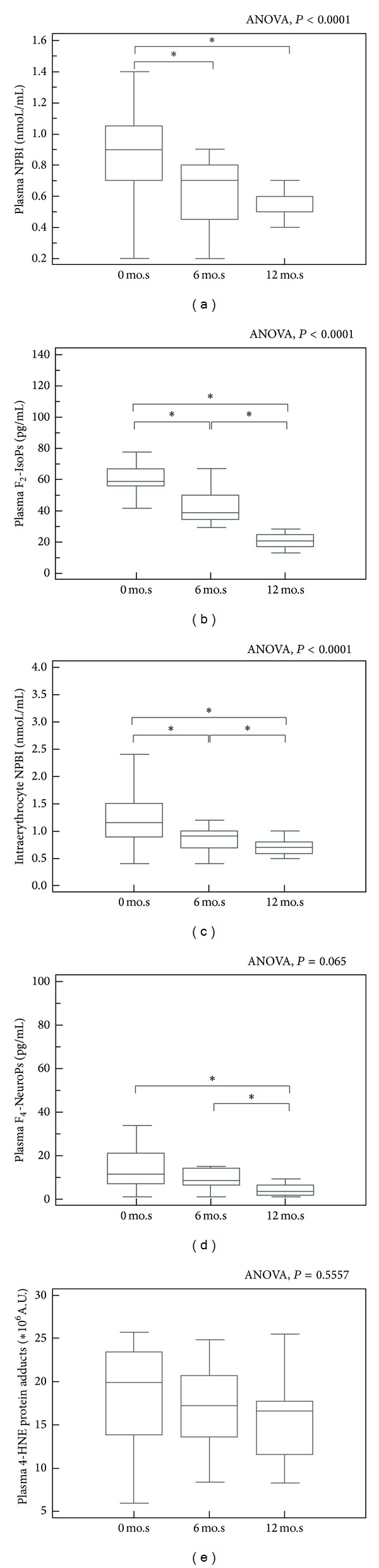
Oxidative stress markers levels (NPBI, plasma F_2_-IsoPs, and F_4_-NeuroPs) are significantly reduced in the *ω*-3 PUFAs supplemented Rett population, as compared to basal values (Panels (a)–(d)). Conversely, no significant changes were observed for 4-HNE PAs values (Panel (e)). *denotes *P* value < 0.05. NPBI: non protein-bound iron; F_2_-IsoPs: plasma free F_2_-isoprostanes; F_4_-NeuroPs: plasma free F_4_-neuroprostanes; 4-HNE protein adducts: 4-hydroxynonenal protein adducts; mo.s: months.

Following 12 months of *ω*-3 PUFAs supplementation, NPBI, plasma F_2_-IsoPs, and F_4_-NeuroPs were significantly reduced as compared to time 0′ values (Figures 2(a)–2(d)). No significant changes were observed for 4-HNE PAs values (Figure 2(e)). Significant differences were already observed in the treated group at time of 6 months for plasma NPBI, intraerythrocyte NPBI, and plasma F_2_-IsoPs.

The correlation matrix for OS markers and myocardial function variables in Rett syndrome following *ω*-3 PUFAs supplementation is reported in Table 2. Plasma F_2_-IsoPs and F_4_-NeuroPs and 4-HNE PAs were found to be inversely related to the left ventricular systolic function parameters.

On the other hand, no significant changes in OS markers were detectable in the untreated RTT patients (data not shown).

In the *ω*-3 PUFAs-supplemented group, clinical severity decreased to 25.5 and 30.1% at 6 and 12 months, respectively (CSS at time zero: 26.2 ± 11.2; CSS at time 6 months: 19.52 ± 8.7; CSS at time 12 months: 18.3 ± 7.8; ANOVA *P* < 0.005; pairwise comparisons time 0 > time 6 months = time 12 months). Conversely, no significant differences in clinical severity were observed in the unsupplemented group of patients (CSS at time zero: 26.0 ± 11.1; CSS at time 12 months: 26.5 ± 10.9; *P* = 0.911). Significant improvements were observed in the areas of attention, breathing abnormalities, muscular tone, ambulation, autonomic dysfunction, and somatic growth.

## 4. Discussion 

Our findings indicate, for the first time, that the subclinical myocardial dysfunction observed in typical RTT can be, at least partially, rescued by 1-year high-dose *ω*-3 PUFAs dietary supplementation. Specifically, *ω*-3 PUFAs appear to reverse all the examined longitudinal systolic parameters (MAPSE, *S*′_lat_, *S*′_sep_ and TAPSE, *S*′_RV_) of the left and right ventricles. Moreover, the improvement in the systolic myocardial function was found to be associated with a marked decrease of OS markers as determined in plasma, whereas no significant changes in the diastolic function were detectable in *ω*-3 PUFAs-treated patients. Taken as a whole, these findings suggest that OS may play a key role in the systolic performance of the RTT myocardium and that it can be at least partially rescued by *ω*-3 PUFAs dietary supplementation.

To date, among the molecular mechanisms potentially underlying the *ω*-3 PUFAs action there are changes in membrane structures and gene expression, direct interactions with ion channels, and alterations in eicosanoid biosynthesis [36]. In particular, EPA and DHA have been reported to compete with arachidonic acid for the conversion by cytochrome P450 enzymes, thus resulting in the formation of alternative, physiologically active, metabolites [37] which could likely mediate some of their beneficial effects [38].

Our current working hypothesis on the beneficial effects of *ω*-3 PUFAs in RTT is that the increased isoprostanes levels in RTT are not simply the effect of the peroxidation of the PUFAs precursors following the attack by radical oxygen species (ROS), but rather the effect of a potential dysregulation of the molecular targets of *ω*-3 PUFAs. Contrary to expectations, the assumed fatty acids are not further oxidized, while the actual endogenous IsoPs production is reduced (the “fatty acid paradox”) together with amelioration of the clinical disease severity [39].

Conceivably, an excess of peroxidation end products from *ω*-6 and *ω*-3 PUFAs would actually imply an excessive consumption of these PUFAs in the cell membranes, thus paving the way for a new perspective on the nutritional horizons in RTT. As RTT girls appear to chronically suffer from oxidation of PUFAs, either *ω*-3 (i.e., DHA/EPA) or *ω*-6 (i.e., AA, AdA), but, at the same time, benefit from *ω*-3 PUFAs supplementation, it can be inferred that these patients would need *ω*-3 PUFAs replacement as a consequence of a persistent PUFAs oxidation within the chronic OS context. On the other hand, it is also possible that, in RTT, the endogenous PUFAs are, for their own nature, more susceptible to the OS as compared to the exogenous ones. Therefore, administered PUFAs may be seen as counteracting this intrinsic defect.

Further research is needed to explore this point, although a very interesting recent report indicates that *ω*-3 PUFAs supplementation, as fish oil, in mice with nonalcoholic fatty liver disease is able to prevent hepatic lipid accumulation and improve lipid metabolism without causing oxidative stress [40]. This report lends further support to our “fatty acids paradox” theory by generalizing it to different abnormal lipid metabolism conditions, either genetic or environmental.

A further critical new piece of research indicates that cholesterol synthesis is impaired in a mutant mouse model in RTT [40]. This latter work strongly indicates that a congenital lipid metabolism error may play a role in the RTT pathogenesis and suggests the use of statins as a potentially valuable alternative treatment for the human disease. In line with this hypothesis, we have previously described an unrecognized hypercholesterolemia in girls affected by the syndrome [41] and pointed out the possibility of an abnormal cholesterol synthesis with a likely partial block in the squalene catabolism due to coexistence of heterozygous mutations in CYP24A1 (OMIM∗126065) or TM7SF2 (OMIM∗603414), which encode the proteins CP24A and ERG24, respectively [18].

Moreover, a promising recent line of research suggests that the beneficial actions of *ω*-3 PUFAs (or their secondary metabolites) could be related to the modulation of an unrecognized subclinical inflammatory status in RTT, a point certainly in need of further exploration, but well fitting with the known anti-inflammatory properties of *ω*-3 PUFAs [26, 42].

On the other hand, a possible explanation for the incomplete rescue of the myocardial dysfunction in RTT could reside in the fact that MeCP2 appears to be involved in myocytes differentiation and maturation [43]. Therefore, the relationship between the MeCP2 protein and heart needs to be further evaluated in the experimental models of the disease.

## 5. Conclusion 

Our data indicate that *ω*-3 PUFAs are able to improve the subclinical biventricular myocardial systolic function observed in typical RTT and that this functional gain is partially mediated through a regulation of the redox balance.

## Figures and Tables

**Table 1:** Phenotypical severity, biometrics, bone densitometry estimates, and 25-hydroxy vitamin D serum levels were comparable between the *ω*-3 PUFAs-supplemented and the untreated Rett patients subgroups.

Variables	Rett syndrome population		*P* value
	*ω*-3 PUFAs supplemented (*n* = 33)	Untreated (*n* = 33)	
Age (years)	13.0 ± 8.6	12.4 ± 9.3	0.7864
Clinical severity score (CSS)	26.2 ± 11.2	26.0 ± 11.1	0.9421
Height (RTT *z*-score for age)^1^	0.078 ± 0.924	−0.025 ± 1.47	0.7344
Body weight (RTT *z*-score for age)^1^	−0.027 ± 1.026	−0.02 ± 1.2	0.9798
Body mass index (RTT *z*-score for age)^1^	−0.30 ± 1.55	−0.40 ± 1.6	0.7973
Head circumference (RTT *z*-score for age)^1^	−0.07 ± 1.23	0.01 ± 0.99	0.7719
Heart rate (bpm)	91 ± 17	93 ± 15	0.6141
Systolic blood pressure (mmHg)	107.8 ± 8.2	107.1 ± 9.3	0.7468
Diastolic blood pressure (mmHg)	70.2 ± 11.6	69.9 ± 8.9	0.9065
Bone densitometry			
AD-SoS (*z*-score for age)	−2.86 ± 1.75	−2.74 ± 1.81	0.7951
BTT (*z*-score for age)	−1.87 ± 1.93	−1.9 ± 1.85	0.9488
Serum 25-OH vitamin D (ng/mL)	45.1 ± 26.1	46.5 ± 25.8	0.8307

^1^ *z*-scores are referred to validated Rett syndrome-specific growth charts [27].

AD-SoS: amplitude-dependent speed of sound; BTT: bone transmission time.

AD-SoS and BTT were evaluated by quantitative ultrasound (QUS) of the distal end of the first phalanx diaphysis of the last four fingers of the hand.

**Table 2:** Correlation matrix for OS markers and myocardial function variables in RTT patients following *ω*-3 PUFAs supplementation.

Echocardiographic variables	Plasma NPBI	Intraerythrocyte NPBI	Plasma F_2_-IsoPs	Plasma F_4_-NeuroPs	Plasma 4-HNE PAs
MAPSE	−0.0363 (0.7654)	0.0707 (0.5608)	**−0.313 (0.0117)**	−0.902 (0.5466)	−0.175 (0.2552)
TAPSE	−0.0045 (0.9710)	−0.0809 (0.5121)	−0.194 (0.1238)	−0.176 (0.2374)	−0.0614 (0.6959)
*S*′_lat_	−0.215^§^ (0.0764)	−0.155 (0.2045)	**−0.258 (0.0392)**	−0.273^§^ (0.0601)	**−0.394 (0.0108)**
*S*′_sept_	−0.0884 (0.4840)	−0.0366 (0.7722)	−0.222 (0.0907)	**−0.340 (0.0240)**	−0.0433 (0.7963)
*S*′_RV_	0.211 (0.1087)	0.126 (0.3434)	0.0292 (0.8338)	−0.0485 (0.7663)	0.0941 (0.5907)
PASP	−0.133 (0.3086)	−0.212 (0.1007)	−0.218 (0.1073)	−0.0481 (0.7712)	0.069 (0.6984)

Data are Spearman's rho correlation coefficients with in brackets *P* values (*N* = 33). Bold characters indicate statistically significant correlations. ^§^indicates statistically non-significant trend. Legend: NPBI: non protein-bound iron; F_2_-IsoPs: free F_2_-isoprostanes; F_4_-NeuroPs: free F_4_-neuroprostanes; 4-HNE PAs: 4-hydroxynonenal protein adducts; MAPSE: mitral annular plane systolic excursion; TAPSE: tricuspid annular plane systolic excursion; *S*′_lat_: peak systolic velocity of lateral mitral annulus; *S*′_sept_: peak systolic velocity of septal mitral annulus; *S*′_RV_: peak systolic velocity of tricuspid annulus of right ventricle; PASP: pulmonary arterial systolic pressure.
